# Editorial: Capillarity and elastocapillarity in biology

**DOI:** 10.1098/rsfs.2025.0020

**Published:** 2025-05-16

**Authors:** David L. Hu, Ho-Young Kim, Dominic Vella

**Affiliations:** ^1^ Woodruff School of Mechanical Engineering and School of Biology, Georgia Institute of Technology, Atlanta, GA, USA; ^2^ Mechanical Engineering, Seoul National University, Seoul, Republic of Korea; ^3^ Mathematical Institute, University of Oxford, Oxford, UK

**Keywords:** capillarity, elastocapillarity, biophysics

## 1. Introduction

This issue of *Interface Focus* features selections of presentations from the Symposium on ‘Capillarity and Elastocapillarity in Biology’ that was held at Seoul National University, South Korea in August 2024. The Symposium was generously supported by the International Union of Theoretical and Applied Mechanics (IUTAM) and was timed to make the most of many people traveling to South Korea for the International Congress of Theoretical and Applied Mechanics (ICTAM) and to celebrate the centenary of IUTAM.

So what are ‘capillarity’ and ‘elastocapillarity’ and why should biologists care about them?

Capillarity has the Latin root *capillaris*, meaning ‘like a hair’. The word refers to the ability of a fluid to spontaneously rise within narrow spaces, especially narrow tubes called ‘capillary tubes’. Capillary rise is such a commonplace phenomenon that it is taken for granted: it allows towels to dry hair, soil to soak up rain and has many consequences in biology. But capillarity is more than just capillary rise: it is all the physical consequences [1] of the surface tension of water, the thin ‘skin’ that separates water from air. Capillarity causes water to form raindrops or bead up when a droplet hits a lotus leaf.

Organisms interact with water throughout their lives, either as the medium in which they live or as a key ingredient for life itself. Capillarity is crucial to life, particularly at the smallest scales, which encapsulates much of the life on the Earth. (The mass of all the ants on the Earth, for example, outweighs all the wild birds and mammals combined.) Capillarity plays a dominant role in the transport of water within plants, the dispersal of fungal spores, water-repellency of arthropods and the fate of bacteria and viruses in respiratory drops.

Given that life has had millions of years to evolve with the physical laws governing capillarity [2], it should not be surprising that organisms have evolved ways not only to live with, but to exploit, surface tension forces. It should also not be a surprise that scientists have taken inspiration from nature to solve engineering challenges. For example, the nanometre-scale cones that texture the wings of cicadas (*Psaltoda claripennis*) have been mimicked in artificial surface coatings that prevent the fogging of glass surfaces [3].

At the same time, a careful understanding of the nature of capillary forces and their impact, or otherwise, on biological organisms can yield new biological insights. One example of this is the resolution of ‘Denny’s paradox’ [4] in which small water striders were once thought to be unable to propel themselves at the water surface. This paradox relied on the understanding that water striders locomote by generating the highly visible waves in their wake—something that juvenile water striders should not be able to do. However, careful experiments and calculations showed that it was not the waves that allow water striders to transfer momentum but rather the vortices beneath the water surface. Paradoxes like this are particularly prevalent in fast biological behaviour.

In the examples given above, the forces due to surface tension are relatively straightforward to understand—the geometry of the organism is fixed. However, another class of problems occurs in biology in which objects are deformed by capillary forces. This is common because many biological materials are soft, and at small length-scales, the forces of surface tension can be large; put another way, the imperative to minimize surface energy can be such that elastic deformation becomes preferable. This regime, termed *elastocapillarity*, also allows for great cross-fertilization between biology and other sciences. For example, such elastocapillarity is key to understanding the mechanical role of flexible hairs on insects and plant leaves, water drinking mechanisms in small birds, shape-morphing of fungi and the water-walking, jumping and diving of aquatic insects. Another phenomenon waiting to be fully understood is Buller’s drop, a small condensed drop that coalesces with another drop, fueling the high-speed ejection of spores from fungi [5].

The study of capillarity has its origins in the 1700s with James Jurin (Jurin’s law for capillary rise), Joseph Plateau (Plateau’s law for the structure of soap films), Thomas Young, Pierre-Simon Laplace (Young–Laplace Equation relating pressure to shape of an interface) and Agnes Pockels (the Pockel’s point, the minimum area a single molecule can occupy in a monomolecular surface film). In modern times, new manufacturing techniques to create synthetic soft surfaces for mimicking structures in nature have expanded the field of capillarity. Similarly, imaging techniques are enabling us to visualize what could only be previously hand drawn. Still, new technology is needed to further yield insight into classical biological problems.

We hope that the examples given above give a sense of the benefits to both biological and engineering sciences of the continued dialogue offered by problems in capillarity and elastocapillarity. This remains an active area of research and made for an exciting Symposium, as we hope the following papers in this special issue demonstrate.

## 2. Capillarity contributions

The surface of water represents a barrier to most organisms. Fish, insects and birds usually stay on their own side of the interface because their methods of respiration and locomotion are tuned to that environment. But as Virgil said, ‘fortune favors the bold’ and indeed several animals venture across the interface to hunt or to escape being hunted.

Jung [6] reviews the many ways in which birds, fish and invertebrates both leap from and dive into the water surface. For the smallest of these creatures, capillarity presents a challenge. Yet, even millimetric copepods can leap to 30 times their body length if they are hunted. Birds dive with sharpened beaks with cone angle as narrow as 10° to pierce the water surface and dive as deep as 22 m below the surface.

Humans can dive as well, and we reduce our drag by pointing our arms above our heads, achieving angles of 30° dictated by our shoulder width and arm length. While Olympic divers try to minimize the deformation of the water surface and splash, certain cultures, such as the Maori, have rituals in which they want to maximize the splash. Rohilla *et al.* [7] study ‘Manu’ diving in which divers actively open up as they dive to generate the largest splash.

At the opposite of the size scale, Kim *et al*. [8] present the respiration of the California blackworm, which lives underwater but breathes air by clinging to the water surface by its tail. Just the tip of its tail is sufficient to support eight times its body weight. The inspiration for this work is a good example of capillarity inspiring biological studies. Vella and Mahadevan’s calculations of the flotation force on spheres and cylinders [9] led Kim *et al.* to calculate the flotation force on organisms like bloodworms, mosquito larvae and snails. Understanding the flotation of these organisms is important because the neuston, the region near the water surface, houses many juvenile organisms. The neuston is increasingly being polluted, causing drastic consequences for the ecosystem.

## 3. Elastocapillarity contributions

While the water surface is prominent for the aforementioned papers, the next category of papers involves three phases: solid, liquid and gas. Moreover, the solids are soft enough that the surface tension can cause deformation.

Li *et al.* [10] demonstrate the importance of this combination of effects on respiration: underwater, gills stay separated due to the surrounding hydrostatic pressure. For water to pass oxygen through gills, the gills must be thin elastic filaments. However, this means that when amphibious animals leave water to go to land, the surface tension forces on the wet surfaces may cause the gills to collapse, reducing their surface area and potentially inhibiting respiration. This may particularly be an issue for amphibious fish such as the mudskipper. Li *et al.* show that the speed of drainage can change the collapsed configuration and hypothesize that amphibious fish may control this leakage rate when exiting the water to regulate respiratory function.

Plants transport water through their xylem by a process of evaporation: water evaporates at the leaves, creating suction that pulls water from the roots, up the stems and to the leaf as the cohesion-tension theory explains. If this process goes as planned, a continuous column of water connects root to stem through the plant’s xylem. However, global warming is leading to drier soils, which can create gas bubbles in the xylem called embolisms. The presence of gas prevents the water from rising properly in the xylem, and it can spread through the hierarchy of veins in the plant. Gauci *et al.* [11] use experiments in polydimethylsiloxane (PDMS) to study how plants can push embolisms along using narrow pliable sections in their xylem.

Ricobelli [12] considers the growth of a spheroid of cells—a model for studying cancer. One way to probe the material properties of the spheroid is to apply a radial cut, like slicing a grape nearly in half. If the material were a liquid, the radial cut would cause the cells to rearrange to form a smaller sphere to reduce surface energy. However, because both surface energy and viscoelasticity are relevant, the spheroid becomes a heart shape. Indeed, surface tension has long been used as a model for the shape of embryos and cells, which like bubbles, are round to minimize surface energy.

Nagoshima *et al.* [13] study the ability of drops to turn wrinkled thin films into folded ones. Here, surface tension of the drop pulls together neighbouring wrinkles to form a deeper groove. Water then drains down these grooves, which become permanent structures in the film. The formation of the drop-cum-wrinkles is also a beautiful art in its own right.

## 4. Conclusion

In addition to the science presented, there are diverse applications of these studies of capillarity. Diving and launching [6] from the water surface have long been of interest to the military in the development of torpedoes and missiles. Drying water drops [13] may be used to form microscopic folded patterns in sheets, from sensors to wicking surfaces mimicking cactus skin. One such application of microscopic folding by a water drop [14] is the generation of DNA nanowires driven by the flow of water from a drop.

Capillarity and elastocapillarity in biology remain fertile ground [15] for research but also art, as can be seen by many of the beautiful photographs and visualizations in this collection. The American Physical Society Gallery of Fluid Motion is an annual photo contest held by the Division of Fluid Dynamics since 1983. Historically, entries involving surface tension, such as drops, splashes and bubbles, have punched above their weight, winning 17% of the prizes across all categories [16]. Thus, capillarity is not just an important tool for understanding biology, but also one of the more enticing jewels in biology’s crown.

## Data Availability

This article has no additional data.
